# Biological response and cytotoxicity induced by lipid nanocapsules

**DOI:** 10.1186/s12951-019-0567-y

**Published:** 2020-01-06

**Authors:** Marzena Szwed, Maria Lyngaas Torgersen, Remya Valsala Kumari, Sunil Kumar Yadava, Sascha Pust, Tore Geir Iversen, Tore Skotland, Jyotsnendu Giri, Kirsten Sandvig

**Affiliations:** 1 Department of Molecular Cell Biology, Institute for Cancer Research, Oslo University Hospital-The Norwegian Radium Hospital, Oslo, Norway; 2 Department of Biomedical Engineering, Indian Institute of Technology, Hyderabad, India; 3 Department of Biosciences, University of Oslo, Oslo, Norway

**Keywords:** Lipid nanocapsules, Breast cancer cells, Ferroptosis, Stress responses

## Abstract

**Background:**

Lipid nanocapsules (LNCs) are promising vehicles for drug delivery. However, since not much was known about cellular toxicity of these nanoparticles in themselves, we have here investigated the mechanisms involved in LNC-induced intoxication of the three breast cancer cell lines MCF-7, MDA-MD-231 and MDA-MB-468. The LNCs used were made of Labrafac™ Lipophile WL1349, Lipoid^®^ S75 and Solutol^®^ HS15.

**Results:**

High resolution SIM microscopy showed that the DiD-labeled LNCs ended up in lysosomes close to the membrane. Empty LNCs, i.e. without encapsulated drug, induced not only increased lysosomal pH, but also acidification of the cytosol and a rapid inhibition of protein synthesis. The cytotoxicity of the LNCs were measured for up to 72 h of incubation using the MTT assay and ATP measurements in all three cell lines, and revealed that MDA-MB-468 was the most sensitive cell line and MCF-7 the least sensitive cell line to these LNCs. The LNCs induced generation of reactive free oxygen species and lipid peroxidation. Experiments with knock-down of kinases in the near-haploid cell line HAP1 indicated that the kinase HRI is essential for the observed phosphorylation of eIF2α. Nrf2 and ATF4 seem to play a protective role against the LNCs in MDA-MB-231 cells, as knock-down of these factors sensitizes the cells to the LNCs. This is in contrast to MCF-7 cells where the knock-down of these factors had a minor effect on the toxicity of the LNCs. Inhibitors of ferroptosis provided a large protection against LNC toxicity in MDA-MB-231 cells, but not in MCF-7 cells.

**Conclusions:**

High doses of LNCs showed a different degree of toxicity on the three cell lines studied, i.e. MCF-7, MDA-MD-231 and MDA-MB-468 and affected signaling factors and the cell fate differently in these cell lines.

## Background

During recent years many new nanoparticles (NPs) have been developed for drug delivery. In most cases the goal is to encapsulate drugs within the NPs, to protect the drug from enzymatic degradation and hopefully to deliver a larger fraction of the drug to the diseased area. The enhanced permeability and retention (EPR) effect of the blood vessels in tumors [1] is able to facilitate transport of NPs to the tumors, as found, e.g. for the first approved NP-based product, Doxil^®^/Caelyx^®^, which contains the cytostatic drug doxorubicin inside liposomes [2, 3].

Accumulation of NPs in tumors based on the EPR effect may also have an effect on the tumor microenvironment by driving the pro-tumorigenic (anti-inflammatory) M2 macrophages into anti-tumorigenic (pro-inflammatory) M1 macrophages [4]. Two recent studies demonstrate how different cellular uptake of NPs into these macrophages may contribute to effects on tumors. The M2 macrophages showed a vigorous uptake by macropinocytosis, whereas this uptake mechanism was virtually inactive in the M1 macrophages [5]. Moreover, it was demonstrated in another study that the M2 macrophages use endocytosis to degrade collagen, thus promoting the growth of solid tumors [6]. Together these effects may, in addition to a longer circulation time of the NP-encapsulated drug, both help to improve the therapeutic treatment with the drug and reduce the systemic toxicity [7, 8].

In the field of nanomedicine there are today a number of studies of NPs for drug delivery that are not biodegradable and may in our opinion never become approved for injections into humans. Thus, it is very important due to safety evaluations that NPs larger than 10 nm (thus being too large to be excreted in urine) are biodegradable, such that the constituents either are degraded into endogenous substances or excreted [9]. Different lipid-based drug-delivery systems such as liposomes, micelles, nanocapsules and nanodiscs [10–13] are thus of interest for use as NPs, as they can be metabolized in the body and several of the components used are endogenous substances. We here report new mechanistic studies performed with lipid nanocapsules (LNCs), which although they have been used for drug delivery in cells and animals models for many years, still need further characterization when it comes to their interaction with cells; for reviews of LNCs and other lipid-based NPs see [11, 13, 14]. The LNCs can be made by different combinations of lipids and surfactants; such substances available as marketed products and approved by different regulatory authorities are listed and summarized in Beloqui et al. [14].

Fluorescent dye labeling of LNCs have been used to study their cellular uptake and in vivo biodistribution in small animals. Nile Red was the most popular fluorescent dye in early studies, until Bastiat et al. [15] in 2013 showed that Nile Red could diffuse out of the LNCs, whereas lipophilic indo-carbocyanines such as DiL (1,1′-dioctadecyl-3,3,3′,3′-tetramethylindocarbocyanine perchlorate) and DiD (1,1′-dioctadecyl-3,3,3′,3′-tetramethylindocarbocyanine 4-chlorobenzenesulfonate) were found to give stable fluorescent labeling of the LNCs. Although many studies are published with drug-loaded LNCs we are aware of only two studies with empty LNCs (i.e. without drugs); both from the same group. Cellular uptake and intracellular transport of LNCs was studied in rat glioma cells [16], and cytotoxicity was measured up to 24 h after adding LNCs to the mouse macrophage-like cells RAW264.7 [17].

In the present study we have investigated the effect of LNCs, made of Labrafac™ Lipophile WL1349, Lipoid^®^ S75 and Solutol^®^ HS15, on the three breast cancer cells lines MCF-7, MDA-MB-231 and MDA-MB-468, which have different properties and represent different types of breast cancer [18]. Although a future goal is to add cancer drugs to these particles, it is an advantage to know how the particles in themselves affect cells. In the present study we have thus investigated the effect of these LNCs without drug as the first step of studying the possibility to use such particles for drug delivery. The combined effect of the nanoparticles and the encapsulated drug may be important both for therapeutic effect and possible side effects. High resolution SIM microscopy demonstrated that the DiD-labeled LNCs end up in lysosomes. We also showed that the LNCs have a reversible inhibitory effect on protein synthesis and on the lysosomal pH. The cytotoxic effect of the LNCs was studied for up to 72 h incubation with particular emphasis on the cellular stress responses, including the generation of reactive oxygen species (ROS), as we recently showed that even small variations in the NPs structure may give different cellular stress responses and mode of cell death [19]. By employing a near-haploid cell line (HAP1) with knockout of specific kinases, we have characterized the pathway involved in the toxic effects of the LNCs.

## Materials and methods

### Materials

Kolliphor^®^ HS 15 (Polyethylene glycol (15)-hydroxystearate, also known as Solutol^®^ HS 15), H_2_O_2_, reduced glutathione (GSH), concanamycin (Con A), N-acetyl cysteine (NAC), DMEM and RPMI 1640 medium, erastin, dimethyl sulfoxide (DMSO), bovine serum albumin (BSA), liproxstatin-1, deferiprone (DFP), Triton X-100, NP-40, sodium deoxycholate, SDS, *n*-octyl β-d-glucopyranoside, saponin, 3-(4,5-dimethyl-2-thiazolyl)-2,5-diphenyl-2*H*-tetrazolium bromide (MTT; cat#M5655), and penicillin/streptomycin (Pen/Strep P4333) were purchased from Sigma-Aldrich Merck (Germany). Labrafac™ Lipophile WL 1349 (medium-chain triglycerides of caprylic (C8) and capric (C10) acids) was a gift from Gattefosse, Germany. Lipoid S-75 (Fat free soybean phospholipids with 70% phosphatidylcholine) was obtained from Lipoid, Germany. DiD’ solid ((1,1′-dioctadecyl-3,3,3′,3′-tetramethylindodicarbocyanine, 4-chlorobenzenesulfonate salt), the BCA (bicinchoninic) protein measuring kit and the DQ-Red BSA assay kit were obtained from Thermo Fisher Scientific; the CellTiter-Glo^®^ Assay was bought from Promega (Madison, WI). For immunoblotting, antibodies against the following molecules were used: eIF2α (#5324), PERK (#5683), phospho-eIF2αSer51 (p-eIF2α, #3398), ATF4 (#11815), GCN2 (#3302); all from Cell Signaling Technology, Danvers, MA and XBP1s (#619502, BioLegend, San Diego, CA), PKR (#ab32052, Abcam, Cambridge, UK). Milli-Q water was freshly prepared from the Millipore Milli-Q Biocell water purification system. All other chemicals were of analytical quality.

### Preparation of LNCs and physicochemical characterization

Preparation of LNCs was based on using the previously reported solvent free phase inversion method [20]. Briefly, Solutol^®^ HS 15, Labrafac™ Lipophile WL1349 and Lipoid^®^ S-75 in the ratio of 64, 31 and 5% (w/w), respectively, were mixed together using magnetic stirrer. Subsequently, 1.8 ml of aqueous sodium chloride solution (10%, w/v) was added and subjected for three heating and cooling cycles from 60 to 90 °C, ensuring that phase inversion temperature passed. At the end of the third cycle (above phase inversion temperature), 5 ml of cold water was added to break the system and formed stable LNCs. The prepared LNC suspensions were stirred under magnetic stirrer for another 5 min. Then, the desired concentration of LNCs was prepared by adding water; the preparations were stored at 4 °C for the further characterization and use. In order to prepare DiD loaded LNCs, 0.03% (w/w of Labrafac) was dissolved in the Labrafac prior to all preparation steps by magnetic stirring for 5 min. Subsequently, the above-mentioned steps were followed to obtain the DiD loaded LNCs (DiD-LNCs).

The hydrodynamic diameter, polydispersity index (PDI) and zeta potential (ζ) of the LNCs was measured at 25 °C in 10 mM phosphate buffered saline, pH 7.4 (PBS) using a Zetasizer Nano ZS (Malvern Instruments Ltd, Worcestershire, UK). The LNC suspensions were diluted to 1 mg/ml with PBS before analysis. Samples were analyzed in triplicate keeping the instrument in automatic mode. To measure the encapsulation efficiency of the DiD in LNCs, DiD-LNCs were passed through a Sephadex column (G-25 fine, Sigma Aldrich) to remove un-encapsulated DiD dye. Then, DiD-LNCs were solubilized using acetone and analyzed by UV–Visible (Jasco, USA) at 645 nm. The amount of DiD was calculated by using a pre-estimated standard curve. The percent encapsulation efficiency was determined by using the following equation. Encapsulation efficiency (%) = (Amount of DiD quantified/Amount of DiD taken) × 100.

### Cell lines

We used three breast cancer cell lines: the MCF-7 (luminal A), the MDA-MB-231, (triple negative; Claudin low), and the MDA-MB-468 (triple negative; basal) [18]. The cells were obtained from ATCC (Manassas, VA) and cultured in RPMI (MDA-MB-231, MCF-7) or DMEM (MDA-MB-468) medium with GlutaMAX TM (Sigma-Aldrich, St Louis, MO), 10% fetal calf serum (FCS), penicillin (100 U/ml) and streptomycin (100 μg/ml) in standard conditions: 37 °C, 100% humidity and the atmosphere being 5% CO_2_ and 95% air.

HAP1 WT and cells lacking each of the eIF2α kinases—denoted ΔHRI, ΔPERK, ΔGCN2, and ΔPKR were kind gifts from Dr. Pavel Ivanov (Brigham and Women’s Hospital, Boston, USA) [21]. The HAP1 cells were cultured in Iscove’s Modified Dulbecco’s Medium (IMDM, Sigma-Aldrich, St Louis, MO) supplemented with 10% FCS, penicillin (100 U/ml), streptomycin (100 μg/ml).

In all experiments, cells in the logarithmic phase of growth were used. The cells were authenticated and periodically tested for Mycoplasma contamination. All cells were seeded 1 day prior to the experiments.

### Transfection of cells with siRNAs

Small interfering RNAs (siRNAs) were introduced into MDA-MB-231 and MCF-7 cells by reverse transfection. The siRNAs were prepared in RPMI and mixed with Lipofectamine RNAiMax (Invitrogen, Carlsbad, CA) before addition to cells (1 × 10^4^ per well) and siRNA (10 nM). The next day, the culture medium was exchanged and 24 h later treatment with LNCs was performed at 37 °C. The following siRNAs (from Ambion Silencer Select siRNAs, Invitrogen, Carlsbad, CA) were used: Silencer Select Negative Control #1 (4390843), siATF4-1 (ID 1702), siATF4-2 (ID 1704), siNrf2-1 (ID9492), siNrf2-2 (ID 9493).

### Cytotoxicity assays

Cytotoxicity was evaluated by using the MTT (3(4,5-dimethylthiazol-2-yl)-2,5-diphenyltetrazolium bromide) assay, by measuring ATP levels using (CellTiter-Glo^®^), protein synthesis (incorporation of [^3^H]leucine), DNA synthesis (incorporation of [^3^H]thymidine), and by studying cell morphology changes in a microscope.

### MTT assay

Cells (1 × 10^4^) in 100 μl culture medium per well were seeded into 96-well microtiter plates with flat-bottomed wells, 24 h before the experiment and then exposed in triplicate to different concentrations of LNCs for 24, 48 and 72 h (5% CO_2_, 37 °C, 100% humidity). At the end of the incubation the cell medium was aspirated and exchanged with 100 μl medium containing a final concentration of 250 μg/ml of MTT. The incubation was continued for 3 h at 37 °C for formation of the formazan-particles, which were dissolved in DMSO with 1% NH_4_Cl. The absorbance was measured at 580 nm (analytical wavelength) and 720 nm (reference wavelength) with a Synergy2 microplate reader (Biosys Ltd, Essex, UK).

### ATP measurements

ATP levels in LNCs-treated cells were quantified the by using the CellTiter-Glo^®^ assay, according to the manufacturer’s procedure. Cells were incubated in 96-well plates with LNCs for 24, 48 and 72 h at 37 °C; thereafter one half of the volume was removed, replaced with an equal volume of the ATP component and gently mixed for 10 min at room temperature. Subsequently, the lysate was transferred to a light-protected 96-well white plate and luminescence measured in a Synergy2 plate reader (Biosys Ltd., Essex, UK). Some experiments were performed in the presence of reactive oxygen scavengers; the cells were then preincubated for 1 h at 37 °C with 5 mM NAC or 10 mM GSH before performing ATP analyses as described above.

### Measurement of protein synthesis

After incubation of the cells with LNCs (0.5 mg/ml) for up to 4 h, protein synthesis was measured. The cells were treated for 10 min at 37 °C in a HEPES-buffered medium with 1 µCi/ml [^3^H]leucine. Then, the medium was removed, and 5% trichloroacetic acid (TCA) was added. This solution was removed 10 min later, and the cells were washed twice with the same solution to remove free radioactive leucine. The precipitated protein was dissolved in 0.1 M KOH, and the radioactivity associated with the cells was measured in a β-counter (MINAXI, TRI-CARB 4000 SERIES, United Technologies, Packard, Meriden, CT).

### Cell proliferation measured by [^3^H]thymidine incorporation

The effect of LNCs on cell proliferation was assessed by [^3^H]thymidine assay. Briefly, cells were plated at a density of 5 × 10^4^ cells/well (using 24 well plates) and incubated for 24 h. LNCs of different concentrations were added and the incubations prolonged for 24 h. After aspirating the media, [^3^H]thymidine (3 μg/ml in serum free media; 75 μCi/ml) was added and incubated for 30 min at 37 °C. The solution was then replaced with 5% TCA (800 µl/well) followed by incubation for 10 min at room temperature to precipitate nucleic acids and proteins. After washing once more with TCA, the TCA solution was aspirated and 0.1 M KOH (200 µl) was added to the wells followed by incubation for 15 min at room temperature to solubilize the precipitated nucleic acids and proteins. The solution was transferred to scintillation vials, mixed with 3 ml of scintillation fluid (Perkin Elmer, USA) and the radioactivity was measured for 60 s using a scintillation counter (Tri-Carb 2100TR, Packard Bioscience, USA).

### Studies using inhibitors of ferroptosis

MDA-MB-231 and MCF-7 cells were pre-incubated for 1 h at 37 °C with either liproxstatin-1 (Liprox, 1 μM) or deferiprone (DFP, 100 μM) before further incubation for 24 h with various concentrations of LNCs, followed by measurements of ATP levels or [^3^H]thymidine incorporation.

### Cellular morphological changes

In parallel with the experiments we performed using the MTT assay, we looked for cellular morphology changes after LNCs treatment by using phase contrast microscopy. Such images were acquired by an Eclipse TS100 microscope (Nikon, Tokyo, Japan) equipped with a 20× objective and a Digital Sight camera (Nikon, Tokyo, Japan).

### Protein measurements

The amount of protein was assayed by the BCA assay as described by the manufacturer. BSA was used as the standard protein.

### Western blotting

For immunoblotting of proteins, 500,000 cells were incubated for up to 8 h at 37 °C and subjected to lysis in 100 μl Laemmli sample buffer (Bio-Rad Criterion, Bio-Rad, Oxford, UK). Subsequently, the lysate was boiled and sonicated to reduce viscosity. For the HAP1 cell lines, cells were washed in cold phosphate buffered saline (PBS; 10 mM phosphate, 0.15 M NaCl, pH 7.4) and lysed by incubating on ice for 10 min in the “lysis buffer” [0.1 M NaCl, 10 mM Na_2_HPO_4_, pH 7.4, 1 mM EDTA, 1% Triton X-100, 60 mM *n*-octyl β-d-glucopyranoside, and a mixture of protease (cOmplete) and phosphatase (PhosSTOP) inhibitors (Roche, Basel, Switzerland)]. The lysate was sonicated and the protein concentration of the cleared lysate was quantified by BCA assay (Thermo Scientific, Waltham, MA). The same concentrations of protein were loaded on the gel for each lysate. SDS-PAGE separation was performed on a 4–20% TGX gel (Criterion, Bio-Rad, Oxford, UK) and proteins transferred onto a 0.2 μm PVDF membrane with Transblot Turbo (Bio-Rad) system. Membranes were blocked by drying followed by overnight incubation with the indicated primary antibodies in 5% (w/v) BSA, and incubated for 35 min with HRP-conjugated secondary antibodies at room temperature. The protein bands were visualized by chemiluminescence detection with SuperSignal West Dura Extended Duration Substrate (ThermoScientific, Waltham, MA) in a ChemiDoc Imaging System (Bio-Rad Laboratories, Hercules, CA) and measured with Bio-Rad Quantity One on a Chemigenius system (Bio-Rad Laboratories, Hercules, CA). The signal intensities were normalized to the loading control.

### Determination of reactive oxygen species production

Intracellular ROS production was recorded by monitoring changes in the fluorescence of chloromethyl derivative of 2′7′-dichlorodihydrofluorescein diacetate (CM-H2DCFDA; Thermo Scientific, Waltham, MA) directly in the monolayer of MDA-MB-231 or MDA-MB-468 cells, seeded onto 96-well plates (1.5 × 10^4^ cells/well) 24 h earlier. The cells were pre-incubated with the dye (10 μM, 45 min, 37 °C) and placed in full fresh medium without phenol red in the presence or absence of 5 mM NAC for 1 h. Subsequently, the cells were treated for 2 h with LNCs or H_2_O_2_ (100 µM) as a positive control. Fluorescence intensity was monitored with Synergy2 plate reader (BioTek, Winooski, VT) with excitation and emission wavelengths of 485 nm and 528 nm, respectively.

### Lipid peroxidation measurement

Changes in lipid peroxidation were assayed by measurements of Bodipy^®^ 581/591 C11 (C11-BODIPY (581/591, Thermo Scientific, Waltham, MA). This lipophilic fluorophore accumulates in the cellular membrane and emits green fluorescence in the presence of lipid ROS. MDA-MB-231 and MDA-MB-468 cells were incubated for 2 h at 37 °C with LNCs; then the cells were washed with PBS and incubated with C11-BODIPY (final concentration 2.5 μM) for 30 min at 37 °C. Subsequently, the stained cells were harvested by Accutase^®^ Cell Detachment Solution (Sigma-Aldrich, St Louis, MO), centrifuged (300×*g* for 10 min at 4 °C) then washed, resuspended in PBS and subjected to flow cytometry analysis. The dye was excited using a 488 nm Ar laser and detected with the FL1 (545 nm) detector on an LSR II Flow Cytometer (BD Biosciences, San Jose, CA). At least 10,000 cells were recorded for each readout.

### Intracellular accumulation of LNCs

Intracellular accumulation was measured by using DID-labeled LNCs and measuring fluorescence with flow cytometry. Cells were seeded in 24-well plates (50,000 cells/well) and cultured for 1 day at 37 °C before measuring the cellular uptake. The LNCs (0.5 mg/ml) were incubated with the cells for 2 h at 37 °C. The cells were thoroughly washed with PBS to remove loosely bound particles. Subsequently, the cells were harvested by Accutase VR Cell Detachment Solution (Sigma-Aldrich, St Louis, MO), pelleted, resuspended in PBS, and subjected to flow cytometry analysis. The dye was excited using a 633 nm (100 mW) solid state red laser and detected with the 660/20 nm bandpass filter detector on Thermo Attune acoustic flow cytometer equipped with a RL1 channel. To demonstrate that the LNC signal reflects true cellular uptake, and not merely cell surface binding of the LNCs, the same experiment was performed at 4 °C when endocytosis is blocked. The cells were pre-incubated for 30 min at 4 °C before adding LNCs and then the cells were incubated at 4 °C for 2 h. The cells were then detached and the fluorescence measured as described above.

### Super-resolution 3D SIM imaging

MCF-7 cells seeded on cover slips were transduced with CellLight™ lysosomes-GFP, BacMam 2.0 reagent according to the manufacturer’s protocol (Thermo Fisher Scientific) in full media for 16 h and subsequently treated with 0.5 mg/ml LNCs for different time periods. The cells were washed in PBS and then fixed in a 4% (w/v) paraformaldehyde solution at room temperature for 15 min. Coverslips were mounted with ProLong Glass (Invitrogen). 3D-SIM imaging was performed on a Deltavision OMX V4 system (Applied Precision) equipped with an Olympus 60× numerical aperture (NA) 1.42 objective, cooled sCMOS cameras and 405, 488, 568 and 642 nm diode lasers. Z-stacks covering the whole cell were recorded with a Z-spacing of 125 nm. A total of 15 raw images (five phases, three rotations) per plane were collected and reconstructed by using SOFTWORX software (Applied Precision) and processed in FIJI, ImageJ and icy software.

### Measurement of binding and endocytosis of ^125^I-labelled transferrin

Transferrin was labeled with ^125^I as described earlier [22]. MCF-7 cells were incubated with LNCs (0.5 mg/ml) at 37 °C for 2 h in cell medium containing 1% FCS. The cells were then washed twice with PBS and serum free HEPES medium (0.5 ml/well) was added, followed by addition of ^125^I-transferrin (40 ng in a total volume of 200 µl; 25,000 cpm/ng). The cells were then incubated for 10 min at 37 °C. Subsequently, the cells were washed and incubated for 1 h on ice with HEPES medium containing 2 mg/ml pronase to remove surface bound transferrin and detach the cells from the wells. After pronase incubation, the medium containing the cells was centrifuged for 2 min before the radioactive contents in the cell pellet (endocytosed transferrin) and in the supernatant (surface bound transferrin) were measured (LKB Wallac 1261 Multigamma). The data are presented as endocytosed transferrin as percent of total cell-associated (endocytosed and surface-bound) transferrin [23].

### DQ-Red BSA assay

Red DQ-Red BSA requires enzymatic cleavage in acidic intracellular lysosomal compartments to generate a highly fluorescent product that can be monitored by flow cytometry. MCF-7 cells were incubated in RPMI media containing LNCs (0.5 and 1 mg/ml) for 2 h at 37 °C. DQ-Red BSA (10 µg/ml) was then added and the incubation was continued for 15 min. The cells were washed twice with PBS and then incubated in the absence or presence of particles for 45 min to ensure that DQ-Red BSA had reached the lysosomes. Subsequently, the cells were harvested with Accutase VR Cell Detachment Solution (Sigma-Aldrich, St Louis, MO), pelleted, resuspended in PBS, and the red-fluorescence of DQ-Red BSA was analyzed by flow cytometry using an LSR II Flow Cytometer (BD Biosciences, San Jose, CA). The dye was excited using a 531 nm (100 mW) solid state red laser and detected with the 610/20 nm bandpass filter. At least 10 000 cells were recorded for each readout.

### Microscopical studies of lysosomes

MCF-7 cells were cultured as described before and seeded on coverslips. The cells were washed with PBS and then fixed in a 4% (w/v) paraformaldehyde solution at room temperature (Sigma Aldrich) for 15 min and permeabilized in 0.05% (w/v) saponin solution supplemented with BSA (0.2% (w/v)) in PBS for 2 min. The cells were then incubated with anti-LAMP-1 primary antibody (Sigma, L1418) diluted 1:200 in saponin blocking buffer for 1 h at room temperature. Then the cells were again washed three times with 5 min incubations. The cells were subsequently incubated for 1 h with secondary Alexa488-donkey-anti-rabbit antibody diluted 1:200, (Jackson, 711-545-152) After a final washing step, the cover slips were mounted in Mowiol supplemented with the nuclear staining reagent Hoechst overnight at room temperature. Detailed analysis of cells was performed by confocal microscopy (Zeiss LSM 780; Carl Zeiss MicroImaging GmbH, Jena, Germany).

### Lysotracker staining measured by flow cytometry

For flow cytometry, cells were grown on 24-well plates and incubated for 2 h in the presence or absence of LNCs (0.5 or 1 mg/ml). Stock solutions (1 mM) of LysoTracker^®^ Red DND-99 (Thermo Scientific, Waltham, MA) were diluted to 75 nM working solution with RPMI culture media and added directly to the MCF-7 cell culture in the presence or absence of the particles. After incubation of cells with LysoTracker working solution for 30 min at 37 °C, cells were rinsed with PBS, harvested by Accutase^®^ Cell Detachment Solution (Sigma-Aldrich, St Louis, MO), centrifuged (300×*g* for 10 min at 4 °C) washed and resuspended in PBS. Subsequently, the flow cytometry analysis was performed. The dye was excited using a 556 nm Ar laser and detected with the FL1 (600 nm) detector on an LSR II Flow Cytometer (BD Biosciences, San Jose, CA). At least 10 000 cells were recorded for each readout and analyzed by FACS Diva (BD Biosciences) software. Concanamycin A (100 nM) was used as the positive control.

### Measurement of cytosolic pH

To estimate the cytosolic pH, MCF-7 cells were washed with RPMI without phenol red and then incubated for 30 min at 37  °C in the same solution, containing 1:1000 dilution of pHrodo™ Green AM Intracellular pH Indicator and 1:100 dilution of PowerLoad™ concentrate (Thermo Fisher Scientific). Next, the cells were washed with RPMI without phenol red, and 2 h incubation with and without LNCs (0.5 and 1 mg/ml) was performed under cell growth conditions, in a medium supplemented with 1 or 10% FCS. Subsequently, the cells were harvested by Accutase^®^ Cell Detachment Solution (Sigma-Aldrich, St Louis, MO), centrifuged (300×*g* for 10 min at 4 °C), washed and resuspended in PBS. A standard curve was established by incubating cells as described above, and after detachment from wells, for 5 min at 37 °C with a series of pH calibration buffers (pH 4.5, 5.5, 6.5, and 7.5) supplemented with 10 µM valinomycin and 10 µM nigericin (Intracellular pH Calibration Kit; Thermo Fisher Scientific). Fluorescence intensity was measured by LSR II Flow Cytometer (BD Biosciences, San Jose, CA), using a 505 nm Ar laser filter and detected with the FL1 530/30 nm bandpass filter. At least 5000 cells were recorded in duplicates.

### Statistical analysis

Mean values ± standard error of the mean (SEM) were calculated for each condition. The statistical significance of the differences was determined by two-tailed unpaired Student’s t-test, with equal or unequal variances, as appropriate; *p < 0.05; **p < 0.01; ***p < 0.001.

## Results

### Physiochemical properties of the LNCs

**Fig. 1:**
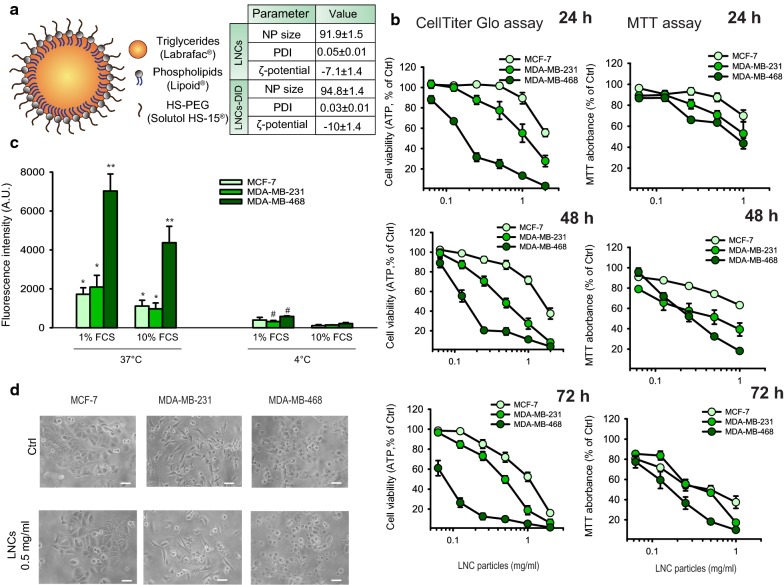
Cytotoxic effect of LNCs on human breast cancer cell lines. **a** Schematic model of the LNCs, including information about their size distribution, PDI and zeta-potential. **b** Viability of MCF-7, MDA-MB-231 and MDA-MB-468 cells was measured with CellTiter-Glo^®^ (ATP levels; left panels) or with the MTT assay (right panels) after incubation of the cells for 24, 48 and 72 h with increasing concentrations of LNCs. The data are shown as percent of untreated control cells and represent mean values ± SEM from four independent experiments. **c** Uptake of LNCs labeled with DID by MCF-7, MDA-MB-231 and MDA-MB-468 cells. Cells were incubated for 2.5 h at either 37 °C or 4 °C with 0.5 mg/ml LNCs using cell growth medium supplemented with 10% or 1% FCS. The graph shows mean values ± SEM from three independent experiments. The p values calculated by comparison of data obtained at 37 °C with data obtained at 4 °C are: *p < 0.05; **p < 0.01. The p-values calculated to compare data obtained with 1% or 10% FCS are: ^#^p < 0.05. **d** Morphological changes in MCF-7, MDA-MB-231 and MDA-MB-468 cells visualized using inverted phase contrast microscopy after treatment of cells for 24 h with 0.5 mg/ml LNCs. The bar shown is 50 µm

The hydrodynamic diameter of the LNCs particles with and without DiD was in the range of 92–95 nm with a narrow size distribution (PDI < 0.05), and the LNCs had a slightly negative charge with a zeta-potential of − 7 to − 10 mV (Fig. 1a). Thus, the physiochemical properties of these LNCs were similar for particles with and without DiD, as also reported by others [24]. The LNC particles were stable in PBS when stored at room temperature for more than 24 h (data not shown). The encapsulation efficiency of the DID in the LNCs was 100 ± 0.5%.

### Cytotoxicity of LNCs in breast cancer cell lines

The cytotoxicity of the LNCs were tested using the human breast cancer cell lines MCF-7, MDA-MB-231 and MDA-MB-468 following incubation at 37 °C for 24, 48 and 72 h. Data obtained with both the MTT assay and ATP measurements (CellTiter-Glo^®^) showed an increased cytotoxic effect with increasing concentrations of LNCs and incubation time in all three cell lines (Fig. 1b). These data show that the MDA-MB-468 cells were most sensitive to the LNCs, and the MCF-7 were the least sensitive of these three cell lines. It should also be noted that the data reveal larger effects of the LNCs when measuring the ATP levels than when using the MTT assay.

To analyze if the cytotoxicity of the LNCs was related to their intracellular accumulation, the cellular uptake of LNCs was estimated after 2.5 h incubation with DID-loaded LNCs in all three cell lines. This was performed at 37 °C in the presence of 1 or 10% FCS, and the results were compared with data obtained at 4 °C when endocytosis is blocked (Fig. 1c). These data correlate well with the cytotoxicity measurements showing the highest uptake of DiD-loaded LNCs by the MDA-MB-468 cells. In parallel, we monitored the morphological changes of these human breast cancer cell lines induced by exposure of the cells to 0.5 mg/ml LNCs for 24 h at 37 °C. The microscopy images in Fig. 1d show a toxic effect on MDA-MB-231 and MDA-MB-468 cells, whereas the MCF-7 cells at this time point looked very similar to the control cells not incubated with LNCs.

### Super resolution 3D SIM imaging shows localization of LNCs in lysosomes

**Fig. 2:**
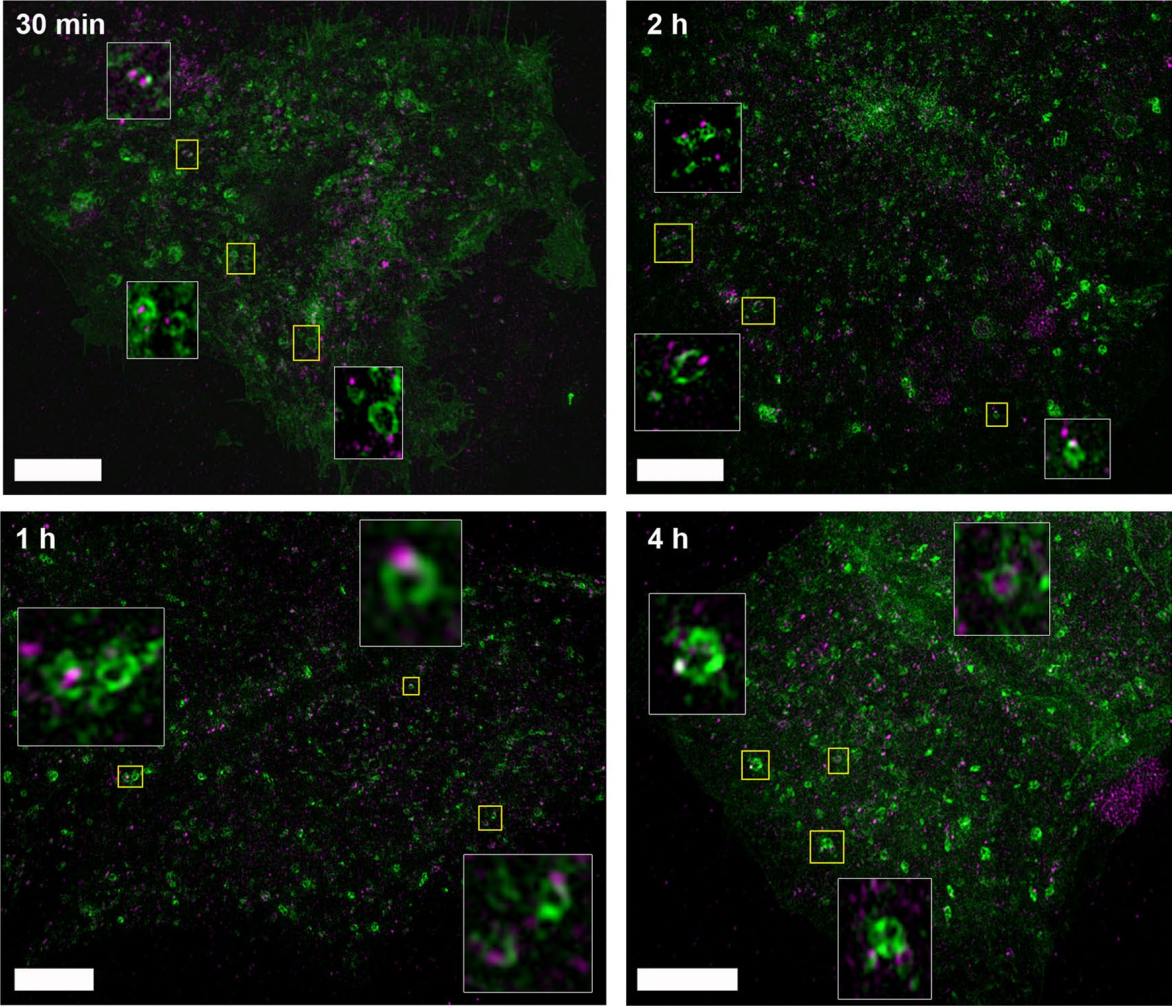
LNCs rapidly localize to lysosomal structures. Super-resolution 3D SIM microscopy of MCF-7 cells treated with DiD-labeled LNCs. The micrographs show the maximum intensity projection of Z-stacks acquired by 2 color super-resolution 3D-SIM with 125 nm increments of MCF-7 cells treated with 0.5 mg/ml DiD-LNCs for the indicated time points. Prior to the treatment with DiD-LNCs, lysosomes were labeled by transduction and overnight incubation with CellLight™ Lysosomes-GFP. Inlays show enlarged pictures of the regions indicated in yellow. LNCs (purple) localize to lysosomal structures (green) already after 30 min. The SIM study was performed once with 10–15 cells analyzed per time point. Scale bars are 5 µm

After endocytic uptake, extracellular material can be transported to lysosomes, which contain enzymes that degrade large molecules into low molecular mass components that can be reused [25]. In order to investigate the intracellular fate of LNCs, we performed super-resolution 3D SIM microscopy. To visualize lysosomal structures, MCF-7 cells were transduced and incubated overnight with CellLight™ Lysosomes-GFP and afterwards incubated with DiD-labeled LNCs for 30 min, 1 h, 2 h and 4 h (0.5 mg/ml). Already after 30 min of incubation DiD-LNCs are found to be associated with lysosomal structures (Fig. 2). Cells take up more LNCs over time, and also more LNCs seem to end up in lysosomal structures in a time-dependent manner. Interestingly, most of the LNCs associated with lysosomes seem to be localized close to the lysosomal membrane, whereas relatively few LNCs can be seen inside the lumen of lysosomes (4 h).

### LNCs rapidly inhibit protein synthesis

**Fig. 3:**
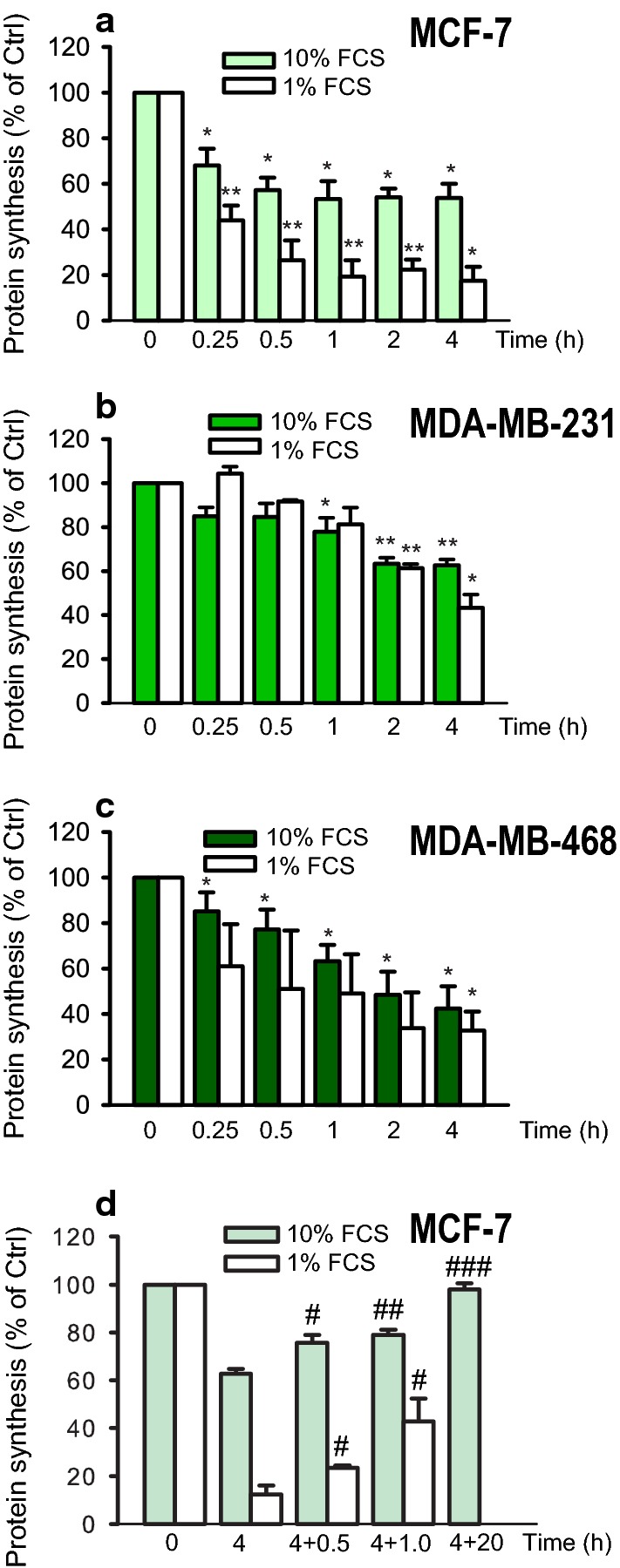
LNCs reduce protein synthesis in breast cancer cells. Protein synthesis was measured in **a** MCF-7 cells, **b** MDA-MB-231 cells and **c** MDA-MB-468 cells. The cells were treated with 0.5 mg/ml of LNCs for the indicated times at 37 °C in culture medium supplemented with 1% or 10% FCS. Protein synthesis was measured by incorporation of [^3^H]leucine for the final 10 min. Data are shown as mean ± SEM from three independent experiments; *p < 0.05; **p < 0.01. **d** Recovery of protein synthesis after removal of LNCs from the medium. MCF-7 cells were treated for 4 h at 37 °C with 0.5 mg/ml LNC; the cell medium was removed, and the cells washed with PBS before fresh medium was added and incubation continued for 0.5, 1.0 and 20 h. The experiment with the longest incubation time was performed with 10% FCS only, as the cells did not tolerate to be incubated that long with only 1% FCS. The data are shown as percent of untreated controls as the mean ± SEM from three independent experiments; ^#^p < 0.05; ^##^p < 0.01; ^###^p < 0.001 for comparison with cells incubated with LNCs for 4 h

Protein synthesis in cells is sensitive to changes in ion composition, pH, ER stress and signaling [26–28], and can provide an early readout of toxic effects. We therefore tested if the LNCs (0.5 mg/ml) had an effect on protein synthesis, as measured by incorporation of [^3^H]leucine, after incubating the cells with LNCs for up to 4 h. As shown in Fig. 3a–c, a reduction in protein synthesis was observed after treatment with LNCs in all cell lines, albeit with slightly different magnitude and kinetics. In MCF-7 cells the protein synthesis was strongly reduced already after 15 min, whereas in MDA-MB-231 cells the reduction was less severe and much slower; a significant reduction was not detectable before 1 h of LNCs treatment. It should be noted that the inhibitory effect of LNCs on protein synthesis after 4 h was reversible, and protein synthesis was back to control levels after a chase period of 20 h in the absence of LNCs (Fig. 3d).

### LNCs activate the integrated stress response

**Fig. 4:**
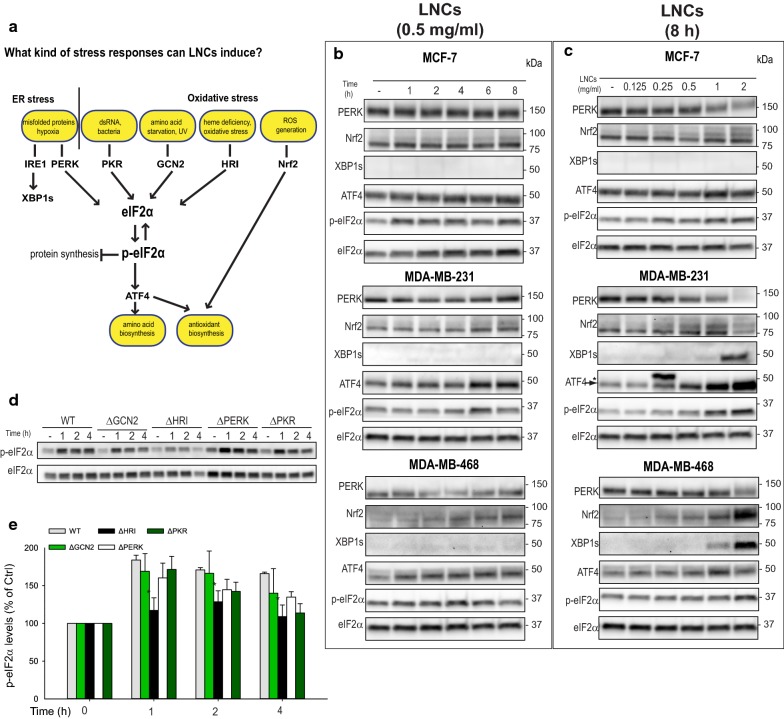
Induction of cellular stress responses by LNCs. **a** Schematic overview of potential cellular stress responses that may be induced by LNCs. The integrated stress response can be triggered by the four kinases GCN2, HRI, PERK and PKR, and be a result of oxidative stress, ER stress, amino acid starvation, unfolded/misfolded proteins, or viral RNA. **b** Cell lysates were prepared from MCF-7 (top), MDA-MB-231 (middle) and MDA-MB-468 (bottom) cells treated with 0.5 mg/ml of LNCs at 37 °C for the indicated time periods. The blots were probed with the indicated antibodies and representative blots are shown. **c** Immunoblots of cell lysates prepared from MCF-7 (top), MDA-MB-231 (middle) and MDA-MB-468 cells (bottom) treated at 37 °C for 8 h with the indicated concentrations of LNCs. **d** HAP1 WT, ΔGCN2, ΔHRI, ΔPERK and ΔPKR cells were treated with 0.5 mg/ml of LNCs for up to 4 h at 37 °C followed by immunoblotting of the cell lysates. One representative experiment is shown. **e** Quantification of data from four independent experiments like the one shown in d. The ratio of p-eIF2α to total eIF2α was normalized to the untreated control cells. Data show mean values ± SEM, *p < 0.05

When cellular homeostasis is altered by stressful conditions, an accumulation of misfolded, ubiquitinated proteins is often observed [27]. We therefore assessed whether the LNC particles induced accumulation of ubiquitinated proteins. When MDA-MB-231 cells were treated with 1 or 2 mg/ml of LNCs for up to 8 h at 37 °C, a large accumulation of ubiquitinated proteins was observed, whereas a similar increase was not observed in the two other cell lines (Additional file 1: Figure S1). This difference may be explained by the more potent and rapid reduction in protein synthesis observed in MCF-7 and MDA-MB-468, which would rapidly reduce the burden of the protein folding machinery. Since accumulation of ubiquitinated proteins may be a consequence of alterations in ER homeostasis (known as ER stress) [29], we asked whether ER stress would be activated by the LNCs. We first assessed the PERK pathway (Fig. 4a), and a weak PERK activation, recognized by an upward shift in PERK migration, was noticed only after 8 h with 2 mg/ml LNCs in MDA-MB-231 cells (Fig. 4b, c). Also the downstream phosphorylation of eIF2α, and accumulation of the transcription factor ATF4 was observed under these conditions in MDA-MB-231 cells (Fig. 4b, c). In contrast, only a minor effect on the PERK pathway was observed in MDA-MB-468 cells, and no effect in MCF-7. When assessing the second ER stress pathway downstream of IRE1 (Fig. 4a) [27], accumulation of XBP1s was observed after prolonged treatment with the highest concentration of LNCs both in MDA-MB-231 and MDA-MB-468 cells (Fig. 4b, c), whereas no effect was observed in MCF-7. Thus, ER stress is induced only by high concentrations of LNCs, and it seems to be most prominently induced in cells where protein synthesis is not rapidly reduced to relieve the ER protein folding machinery.

The PERK pathway is part of the integrated stress response (ISR) that is activated by several external factors such as oxidative stress, proteasomal inhibition, amino acid starvation, ER stress or viral infection [28]. The ISR is regulated by the four kinases PERK, GCN2, PKR and HRI, and activation of these kinases will lead to phosphorylation of eIF2α and trigger ATF4 accumulation and translocation to the nucleus (Fig. 4a). In order to study the involvement of these kinases in LNC-induced stress responses, we took advantage of HAP1 cells with CRISPR/Cas9-mediated knockout of each eIF2α kinase [21]. Treatment of HAP1 wild type cells with LNCs induced a rapid and potent phosphorylation of eIF2α, and a similar induction was observed in ΔPERK, ΔGCN2, or ΔPKR cells (Fig. 4d). However, in ΔHRI cells the phosphorylation of eIF2α upon LNC treatment was significantly reduced (Fig. 4e). HRI is known to be induced downstream of oxidative stress [28, 30], which may indicate that LNCs alter cellular redox homeostasis. Interestingly, and in support of this, treatment with LNCs led to accumulation of the redox sensitive transcription factor Nrf2 in all the three breast cancer cell lines (Fig. 4b, c).

### LNCs affect redox homeostasis in breast cancer cell lines

**Fig. 5:**
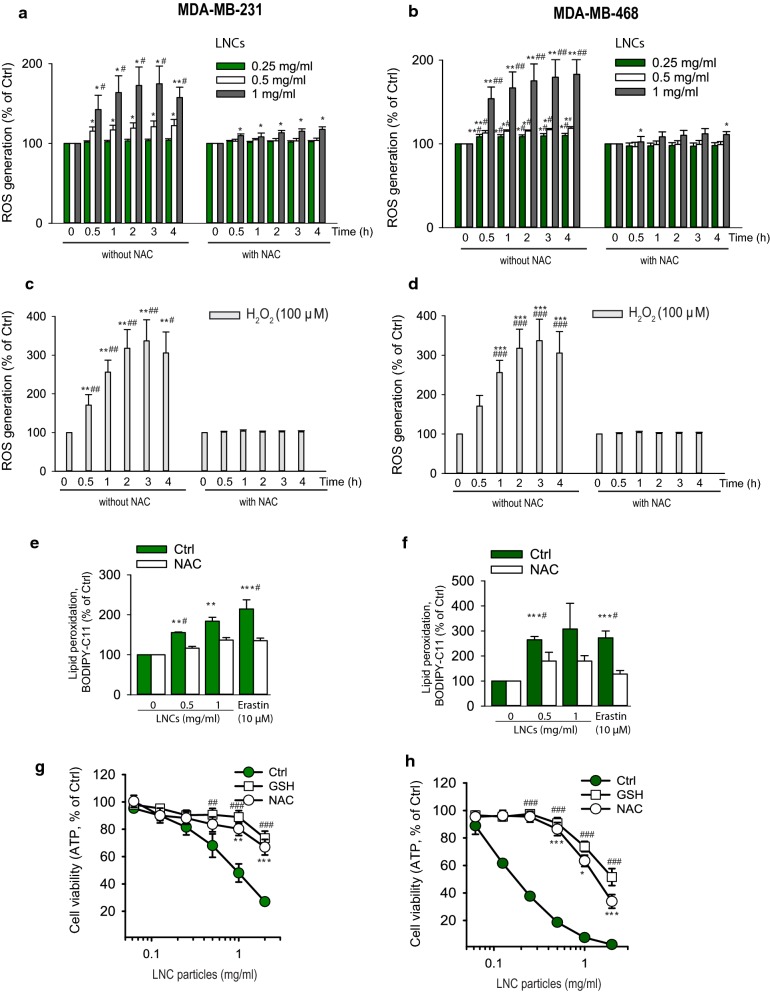
ROS production mediates cytotoxicity of LNCs. Data for MDA-MB-231 are shown on the left side of the figure and data for MDA-MB-468 are shown on the right side of the figure. **a**, **b** Cells were incubated for up to 4 h 37 °C with the indicated concentrations of LNCs in the absence or presence of the antioxidant NAC, and ROS production was measured using the fluorescence probe CM-H_2_DCFDA. Bars represent mean values ± SEM from three independent experiments. *p < 0.05; **p < 0.01; ***p < 0.001 for comparison with data obtained in the absence of LNCs and ^#^p < 0.05; ^##^p < 0.01; ^###^p < 0.001 for comparison with cells preincubated with NAC. **c**, **d** Effect on ROS production after incubation of the cells at 37 °C with 100 µM H_2_O_2_ as a positive control. Bars represent mean values ± SEM from four independent experiments. *p < 0.05; **p < 0.01; ***p < 0.001 for comparison with data obtained in the absence of H_2_O_2_ and ^#^p < 0.05; ^##^p < 0.01; ^###^p < 0.001 for comparison with cells preincubated with NAC. **e**, **f** Changes of lipid peroxidation in MDA-MB-231 and MDA-MB-468 cells following treatment of cells for 2 h at 37 °C with LNCs (0.5 and 1.0 mg/ml). Lipid peroxidation was estimated by flow cytometry after staining of cells with BODIPY-C11 for 30 min. Bars represent mean values ± SEM from four independent experiments; *p < 0.05; **p < 0.01; ***p < 0.001 for comparison with data obtained in the absence of LNCs and ^#^p < 0.05; ^##^p < 0.01; ^###^p < 0.001 for comparison with cells preincubated with NAC. **g**, **h** Cell viability of MDA-MB-231 and MDA-MB-468 measured using the CellTiter-Glo^®^ assay (ATP measurements) after 24 h treatment of cells with varying concentrations of LNCs in the absence or presence of the free radical scavengers NAC (5 mM) and GSH (10 mM). The graphs show mean values ± SEM from at least three independent experiments; *p < 0.05; **p < 0.01; ***p < 0.001 for comparison of cells preincubated with NAC. ^##^p < 0.01; ^###^p < 0.001 for comparison of cells preincubated with GSH (10 mM)

Since both HRI and Nrf2 seemed to be implicated in LNC-induced stress responses, we tested whether these particles induce formation of free radicals. ROS production was determined by using the fluorescent probe CM H_2_DCF-DA. LNC-treated cells displayed an increase in ROS in a time-dependent manner and reached maximal level at 2 h of incubation both in MDA-MB-231 as well as in MDA-MB-468 cells (Fig. 5a, b). At this time point, the level of ROS was increased by about 74% (MDA-MB-231) and 80% (MDA-MB-468) in comparison with the untreated control cells. Almost complete inhibition of ROS accumulation was observed in cells pretreated with NAC, which is a precursor of GSH, the main reducing agent in the cells. As a control, treatment with 100 µM H_2_O_2_ was used (Fig. 5c, d). Formation of ROS is also associated with lipid peroxidation and membrane damage. To test whether the LNCs induced lipid peroxidation, we employed the fluorescent fatty acid analogue Bodipy^®^ 581/591 C11 and erastin as a positive control. Indeed, lipid ROS was induced after 2 h incubation with LNCs both in MDA-MB-231 and MDA-MB-468 cells and NAC could counteract this induction (Fig. 5e, f).

In line with LNC-induced accumulation of both free ROS and lipid ROS, the cytotoxicity of LNC particles (measured as reduced levels of ATP) was potently reversed by co-treatment of MDA-MB-231 and MDA-MB-468 cells with the antioxidants NAC and GSH (Fig. 5g, h and Additional file 1: Figure S3). Thus, an excess of GSH or NAC strongly improved viability of LNC-treated cells suggesting involvement of oxidative stress. This hypothesis was also supported by microscopic examination of cell morphology after treatment with LNCs (Additional file 1: Figure S3). Noticeable changes such as cell shrinkage and blebbing from the plasma membrane were detected in MDA-MB-231 and MDA-MB-468 cells exposed to LNCs. In cells preincubated with GSH and NAC, a reduction of the morphological changes was observed. In contrast to the results described above, GSH and NAC did not have any significant protective effect on the ATP level in MCF-7 cells after 24 h (our unpublished data).

### LNCs induce ferroptosis in MDA-MB-231 cells

**Fig. 6:**
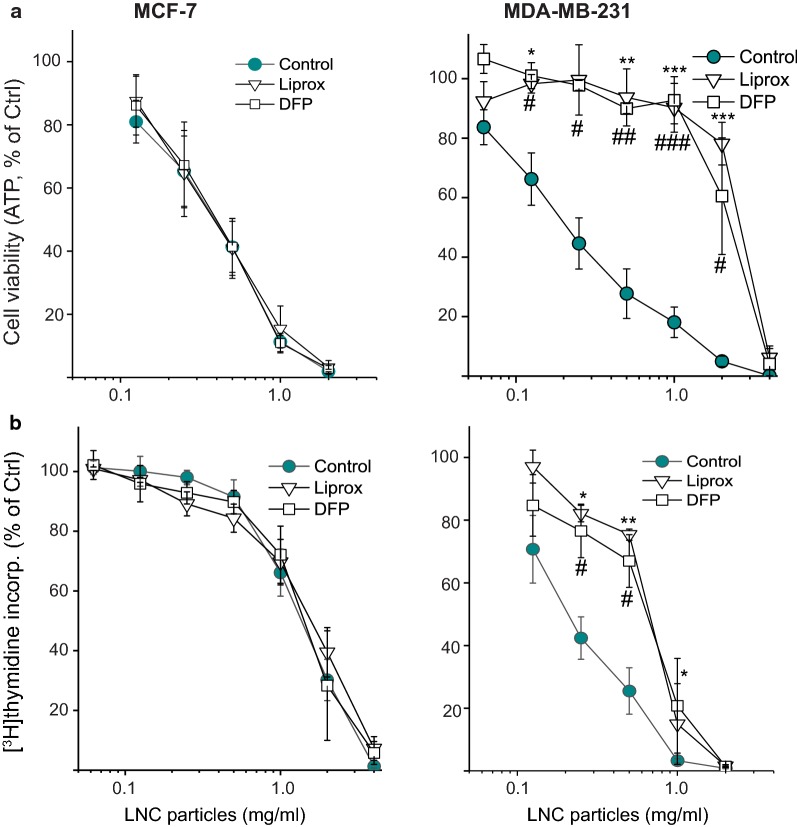
Ferroptosis inhibitors protect MDA-MB-231 cells, but not MCF-7 cells to LNC-induced toxicity. Cytotoxicity was assessed by measuring ATP levels (**a**) and [^3^H]thymidine incorporation (**b**) of MCF-7 cells and MDA-MB-231 cells treated with varying concentrations of LNCs for 24 h in the presence or absence of liproxstatin-1 (Liprox, 1 μM) or deferiprone (DFP, 100 μM). The data are shown as percent of control for each cell line not treated with LNCs. The data are shown as percent of control for each cell line not treated with LNCs. The graphs show mean values ± SEM from three independent experiments. *p < 0.05; **p < 0.01; ***p < 0.005 for comparison of cells treated with Liprox, and ^#^p < 0.05; ^###^p < 0.005 for comparison of cells treated with DFP

MDA-MB-231 cells have been shown to be vulnerable to ROS generation and concomitant lipid peroxidation, due to their lack of the membrane-associated glutathione-dependent lipid hydroperoxidase (GPX4) [31]. In contrast, MCF-7 cells overexpress GPX4. Ferroptosis is a recently discovered iron-dependent type of regulated necrosis associated with accumulation of ROS, excessive lipid peroxidation and loss of GSH [32]. Lipophilic small-molecule antioxidants have been shown to rescue cells from ferroptosis [33]. To test whether the MDA-MB-231 and MCF-7 cells undergo ferroptosis upon LNC-induced cytotoxicity, we treated the cells with LNCs in the presence of ferroptosis inhibitors. By measuring ATP levels and thymidine incorporation, we found that the lipophilic antioxidant liproxstatin-1 and iron chelation with deferiprone significantly rescued MDA-MB-231 cells from the LNC-induced toxicity but had no significant effect in MCF-7 cells (Fig. 6).

### The cytotoxicity of LNCs is higher in ATF4 and Nrf2- depleted breast cancer cells

**Fig. 7:**
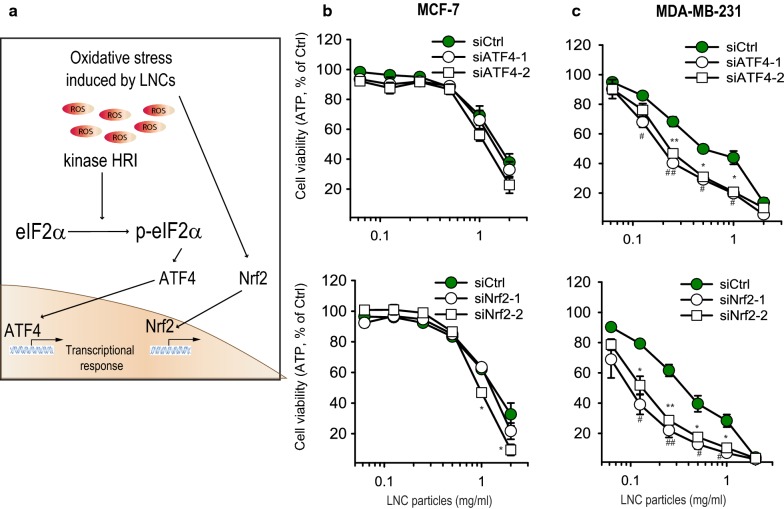
Knock-down of the transcription factors ATF4 and Nrf2 sensitizes the cells to LNC-induced toxicity. **a** Schematic model of the relationship between intracellular redox imbalance after treatment with LNCs and the accumulation of ATF4 and Nrf2 in the nucleus. **b**, **c** Cell viability assessed by measuring ATP levels in MCF-7 and MDA-MB-231 cells depleted for either ATF4 or Nrf2 for 48 h and then treated for 24 h at 37 °C with varying concentrations of LNCs. The data are shown as percent of control for each cell line not treated with LNCs. *p < 0.05; **p < 0.01, for comparison of cells depleted for ATF4. ^##^p < 0.01; ^###^p < 0.001 for comparison of cells depleted for Nrf2

As described above, oxidative stress was found to contribute to LNC cytotoxicity towards the breast cancer cells used in this study (Fig. 7a). Moreover, the LNC-induced phosphorylation of eIF2α seemed to largely depend on the HRI kinase (Fig. 4d, e), which is known to respond to oxidative stress [28, 30]. In addition, both ATF4 and Nrf2 were accumulated in LNC-treated MDA-MB-231 cells (Fig. 4b, c). Since ATF4 and Nrf2 mediate the synthesis of antioxidant proteins [34], we examined whether these two transcription factors contribute to a prosurvival response in LNC-treated cells. We chose cell lines with different resistance to the LNCs: the highly sensitive MDA-MB-231 and the more resistant MCF-7 (Fig. 1b). Interestingly, and in line with our previous study [19], depletion of ATF4 or Nrf2 by siRNA in MDA-MB-231 cells, potently increased the cytotoxicity of LNCs (Fig. 7b, c). This suggests that ATF4 and Nrf2 contribute to pro-survival responses that prevent or delay cytotoxicity in LNC-treated cells. In contrast, depletion of ATF4 or Nrf2 hardly alters LNC cytotoxicity in MCF-7 cells (Fig. 7b, c). Thus, the apparent resistance towards LNC-induced toxicity displayed by MCF-7 cells does not seem to depend on ATF4 or Nrf2.

### LNCs affect endocytosis and lysosomal pH

**Fig. 8:**
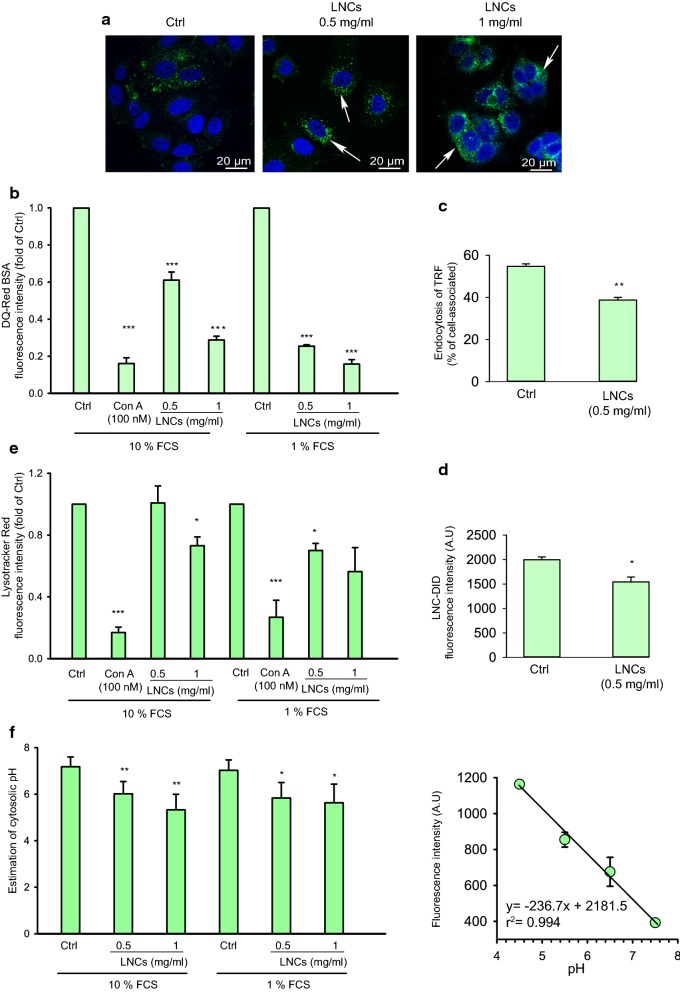
Effect of LNCs on endocytosis and intracellular transport. Details as described in Materials and Methods. **a** Representative fluorescence micrographs of MCF-7 cells treated with 0, 0.5 and 1 mg/ml LNCs in full growth culture medium for 4 h at 37 °C before fixation. The cell nucleus was stained with Hoechst (blue) and lysosomes were stained with anti‐LAMP1 (green). The arrows indicate apparently larger and/or aggregated lysosomes. The scale bar represents 20 μm. **b** Measurements of DQ-Red BSA fluorescence after 2 h incubation at 37 °C of MCF-7 cells with 0.5 and 1.0 mg/ml of LNCs; the v-ATPase inhibitor Concanamycin A (100 nM) was added at the same time as the LNCs. The experiments were performed in cell growth medium supplemented with 10% or 1% FCS. The median fluorescence intensity of DQ-Red BSA was measured by flow cytometry. **c** Endocytosis of ^125^I-labeled transferrin in MCF-7 cells incubated with and without 0.5 mg/ml LNCs for 2 h at 37 °C. The error bars show mean ± SEM from three independent experiments.*p < 0.05. **d** Uptake of LNCs stained with DID after incubation as in c. The results are shown as the fluorescence intensity measured in 10,000 viable cells by flow cytometry. *p < 0.05. **e** The staining of lysosomes of MCF-7 cells by Lysotracker Red^®^ after 2 h incubation at 37 °C with LNCs (0.5 and 1 mg/ml) in medium supplemented with 1% and 10% FCS as described in methods. The fluorescence intensity of Lysotracker Red^®^ was detected by flow cytometry (10,000 cells were collected). **f** Cytosolic pH was estimated after 2 h incubation in the absence and presence of LNCs as described in “Materials and methods”. The resulting cytosolic pH is shown to the left, and the standard curve to the right. Error bars for the cytosolic pH show mean ± SEM; n = 6. Error bars for the standard curve show mean ± SD; n = 2

Endocytosed LNCs were shown to be present in lysosomes of MCF-7 cells after 30 min incubation (Fig. 2). Moreover, when the LNC-treatment was prolonged to 2 h, the lysosomes of LNC-treated cells seemed to be larger and/or aggregated than those in untreated cells (Fig. 8a). One of the first markers indicating decreased functionality of lysosomes is a reduction in the activity of lysosomal proteases [35]. To assess lysosomal functionality, we employed the BSA conjugate DQ-Red BSA, which upon proteolysis in the acidic environment of the lysosomes, releases brightly fluorescent protein fragments [36]. Upon treatment of MCF-7 cells with LNCs the released fluorescence intensity from DQ-Red BSA was strongly reduced (Fig. 8b), suggesting reduced lysosomal cleavage activity and/or a strong reduction in uptake of the probe into lysosomes. Notably, we observed that the inhibitory effect was smaller if the particles were absent during the 15 min incubation with the probe and the 45 min chase to allow transport of the probe to lysosomes (Additional file 1: Figure S4a) suggesting that a change in lysosomal pH is the main factor.

To assess a potential effect of LNCs on endocytosis, we measured the uptake of transferrin (Tf), which is internalized by clathrin-dependent endocytosis [37]. As shown in Fig. 8c, 2 h preincubation with LNCs reduced the uptake of transferrin by ~ 30%. Furthermore, pretreatment with LNCs did not only reduce uptake of transferrin as a cargo, but also reduced the uptake of DiD-labeled LNCs (Fig. 8d). Since inhibition of lysosomal proteases can be related to an increase in lysosomal pH, we also measured the accumulation of LysoTracker™ Red DND-99, which is freely permeant to cell membranes and retained after protonation in acid lysosomes [38]. In MCF-7 cells treated with LNCs (1 mg/ml) the fluorescence of LysoTracker™ was reduced after 2 h incubation with LNCs, and the effect was even stronger in the presence of 1% FBS (Fig. 8e). We did not observe any effect on lysosomal pH when the particles were absent during the incubation with probe (Additional file 1: Figure. S4b), suggesting a reversible effect on lysosomal pH. These data are in agreement with the finding that removal of LNCs from the culture medium may allow reversal of toxic effects (Fig. 3d) after relatively short time of incubation with LNCs.

An increase in lysosomal pH caused by inhibition of the v-ATPase or permeabilization of the lysosomal membrane, may contribute to an acidification of the cytosol. Furthermore, hydrolysis of LNCs leading to release of fatty acyl groups which may pick up protons, penetrate the lysosomal membrane and dissociate (loose protons) in the cytosol, may contribute to lower pH in the cytosol and a higher pH in the lysosomes. We therefore tested whether cytosolic pH was affected by incubations with LNCs. As shown in Fig. 8f, this was indeed the case. After 2 h treatment with 0.5 and 1 mg/ml LNCs, the pH in the cytosol was found to be somewhat reduced (Fig. 8f). However, although this is statistically significant, the values shown in the figure are associated with some uncertainty.

## Discussion

In the present study we used three breast cancer cell lines, MCF-7, MDA-MB-231 and MDA-MB-468, to study cellular stress responses and the cytotoxic effect of empty LNCs. MDA-MB468 was the most sensitive cell line and MCF-7 the least sensitive cell line at all time points measured (24, 48 and 72 h) using both ATP measurements and the MTT assay, and the effect was largest when measuring ATP. This demonstrates that it is useful to include different test systems when investigating the effect of NPs on cells [19], and the differences here observed between the two test systems are similar to what we have published previously for other NPs [39]. This different sensitivity for the three cell lines against LNCs was in contrast to the measured inhibition of protein synthesis, where MCF-7 seems to be the most sensitive cell line, indicating that the rapid initial effect on protein synthesis is not the most important factor leading to cell death. It is not clear why the effect on protein synthesis is so rapid; it was observed already after 15 min incubation. It has been reported that signaling from PERK and HRI inhibits protein synthesis [27, 28], but our data indicate that the effect on protein synthesis is faster than that observed for these signaling molecules. In MCF-7 and MDA-MB-231 cells ISRIB, an inhibitor of downstream effects of eIF2α had no protective effect against protein synthesis inhibition (data not shown). It should also be noted that the effect of protein synthesis was reversible.

Importantly, we observed acidification of the cytosol following addition of LNCs. As mentioned in Results, hydrolysis of the lipids of the LNCs may contribute to alkalinization of the lysosomes and acidification of the cytosol, which may affect protein synthesis. A low cytosolic pH has been shown to inhibit endocytosis and have a main effect on clathrin-mediated endocytosis [40]. Thus, this change in pH may be involved in the measured LNC-induced inhibition of transferrin endocytosis.

Several of the effects on cells were investigated in the presence of either 1% or 10% FCS as this may provide the LNCs with different protein coronas and serum growth factors may play a role for signaling in cells. As expected the highest concentration of FCS gave less cellular uptake of the LNCs and the high FCS concentration also reduced several cellular effects of the LNCs as shown in Figs. 3 and 8.

It has previously been reported that toxicity of LNCs to different cell lines (HaCaT and RAW246.7) is mainly due to the non-ionic Solutol^®^ HS15 components [17, 41]. According to previous reports, it is not clear why Solutol^®^ HS15 has a toxic effect on cells [17, 41]. We observed a reduced fluorescence of Lysotracker in cells following addition of LNCs, which is in agreement with the report that treatment of rat glioma cells with LNCs resulted in less uptake of Neutral Red in lysosomes [16]. It should be noted that our high-resolution SIM 3D imaging revealed the LNCs to be localized close to the membrane in lysosomes. The molecule DQ-Red BSA gives a red fluorescence when cleaved by lysosomal proteases, and in agreement with the finding that there is a slight reduction of endocytic uptake and an increased lysosomal pH, this probe was to a small extent processed to fluorescent products after LNC treatment.

In agreement with the idea that reactive oxygen species (ROS) may be involved in the loss of cell viability following incubations with LNCs, is the finding that GSH (a main antioxidant in cells) and NAC (a precursor of GSH) protect the MDA-MB-231 and MDA-MB-468 cells against the LNC-induced cell death. It should be noted that an effect on lipid peroxidation was observed already after 2 h incubation at 37 °C with 0.5 mg/ml LNCs. Moreover, in the MCF-7 cells treatment with GSH and NAC did not give a significant protective effect against LNC-induced cell death. This is in agreement with Theodossiou et al. [29], reporting a high level of GPX4 in MCF-7 cells. They also found that an 80% reduction of the intracellular GSH level had no sensitizing effect on ROS-induced toxicity in MCF-7 cells. This increased resistance to LNC-induced cell death may be related to the more efficient GPX4-mediated antioxidant defense in MCF-7 than in MDA-MB-231 [31].

As described, we found that LNCs were more than 10-fold more toxic to MDA-MB-231 cells than to MCF-7 cells, and ferroptosis was identified as the LNC-induced cell-death mechanism in MDA-MB-231 cells but not in the MCF-7 cells. The transcription factors Nrf2 and ATF4 were upregulated to varying extent following addition of LNCs, and one may speculate that this is a protective response when cells are exposed to LNCs. Knock-down of these proteins in MDA-MB-231 cells had a strong sensitizing effect to the LNC-induced toxicity, whereas there was hardly any effect on the MCF-7 cells, results supporting the view that upregulation of transcription factors occurs to protect the cells. In conclusion, LNCs can be toxic to cells at high concentrations, but affect cell physiology in a cell-type dependent manner.

## Conclusions

LNCs are promising nanoparticles for drug delivery. To optimize their further development and use one needs to understand their mechanism of action on cells. We here demonstrate that high doses of LNCs showed a different degree of toxicity on the three breast cancer cell lines MCF-7, MDA-MB-231 and MDA-MB-468. The LNCs ended up in lysosomes and induced both increased lysosomal pH, acidification of cytosol and a rapid inhibition of protein synthesis. The LNCs affected signaling factors and the cell fate differently in these cell lines, and our studies revealed that MDA-MB-468 was most sensitive and MCF-7 the least sensitive of the three cell lines. The LNCs were able to induce generation of reactive free oxygen species and lipid peroxidation, and Nrf2 and ATF4 were found to have a protective role against the LNCs in MDA-MB-231 cells, as knock-down of these factors sensitized the cells to the LNCs. This is in contrast to what we observed with MCF-7 cells where the knock-down of these factors only had a minor effect on the toxicity of LNCs. Furthermore, inhibitors of the newly described ferroptosis death mechanism provided a large protection against LNCs in MDA-MB-231 cells, but not in MCF-7 cells. These results demonstrate that the mechanism of action of the LNCs is highly cell type dependent and that their action should be tested specifically on the cancer types one wants to treat. These observations provide new data of importance for the use of LNCs for drug delivery.

## Supplementary information

**Additional file 1.** Additional figures.

## Data Availability

All data generated or analyzed during this study are included in this published article and its additional file.
