# Supplementary material for: Biological response and cytotoxicity induced by lipid nanocapsules
Source: J Nanobiotechnology. 2020 Jan 6;18:5. doi: 10.1186/s12951-019-0567-y (PMC6943936; doi:10.1186/s12951-019-0567-y)
Supplement: Supplementary file 1 — Additional file 1. Additional figures. [file 12951_2019_567_MOESM1_ESM.docx]

**Additional file 1**

**Biological response and cytotoxicity induced by lipid nanocapsules**

Marzena Szwed^1^, Maria Lyngaas Torgersen^1,*^, Remya Valsala Kumari^2,*^, Sunil Kumar Yadava^2,*^, Sascha Pust^1^, Tore Geir Iversen^1^, Tore Skotland^1^, Jyotsnendu Giri^2,#^ and Kirsten Sandvig^1,3,#^

^1^Department of Molecular Cell Biology, Institute for Cancer Research, Oslo University Hospital - The Norwegian Radium Hospital, Oslo, Norway

^2^Department of Biomedical Engineering, Indian Institute of Technology, Hyderabad, India

^3^Department of Biosciences, University of Oslo, Oslo, Norway

^*^ Contributed equally

^#^ Corresponding authors: [ksandvig@radium.uio.no](mailto:ksandvig@radium.uio.no) and [j.giri@iith.ac.in](mailto:j.giri@iith.ac.in)

**
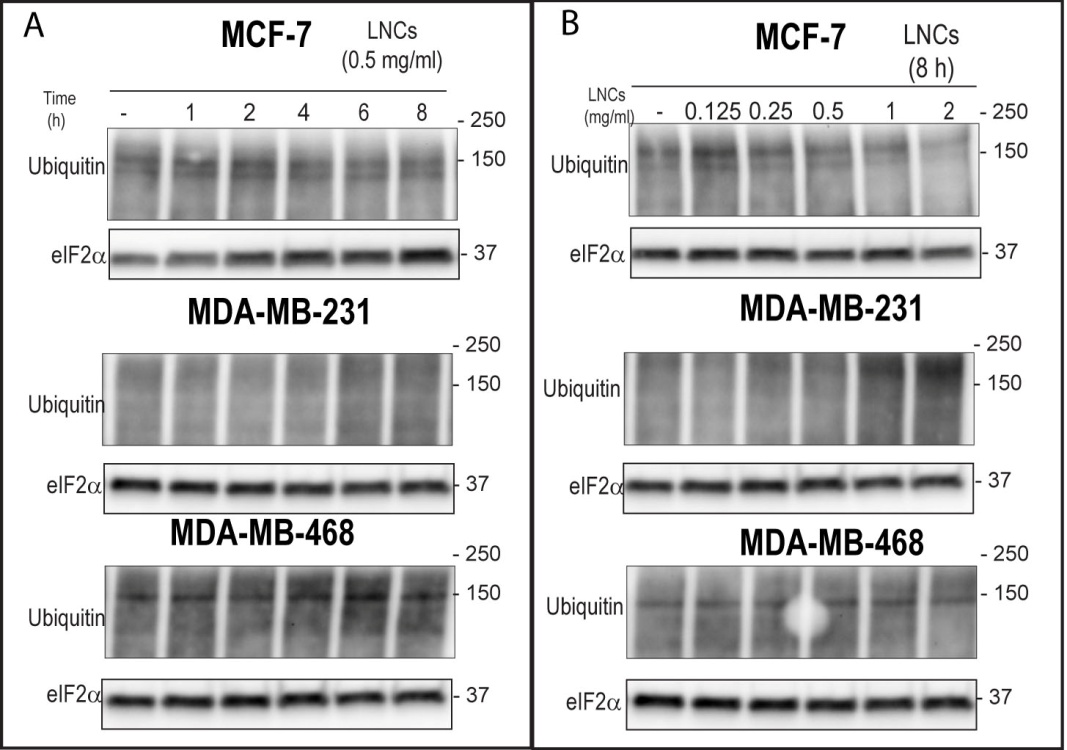
**

**Figure S1. a** Immunoblots of ubiquitin measured in cell lysates prepared from MCF-7 (top), MDA-MB-231 (middle) and MDA-MB-468 (bottom) cells treated at 37°C for the indicated time periods with 0.5 mg/ml of LNCs. The blots were probed with the indicated antibodies and representative blots are shown. **b** Immunoblots of ubiquitin in cell lysates prepared from MCF-7 (top), MDA-MB-231 (middle) and MDA-MB-468 cells (bottom) treated with the concentrations of LNCs shown for 8 h at 37°C. The representative blots were obtained similarly as in a.

**
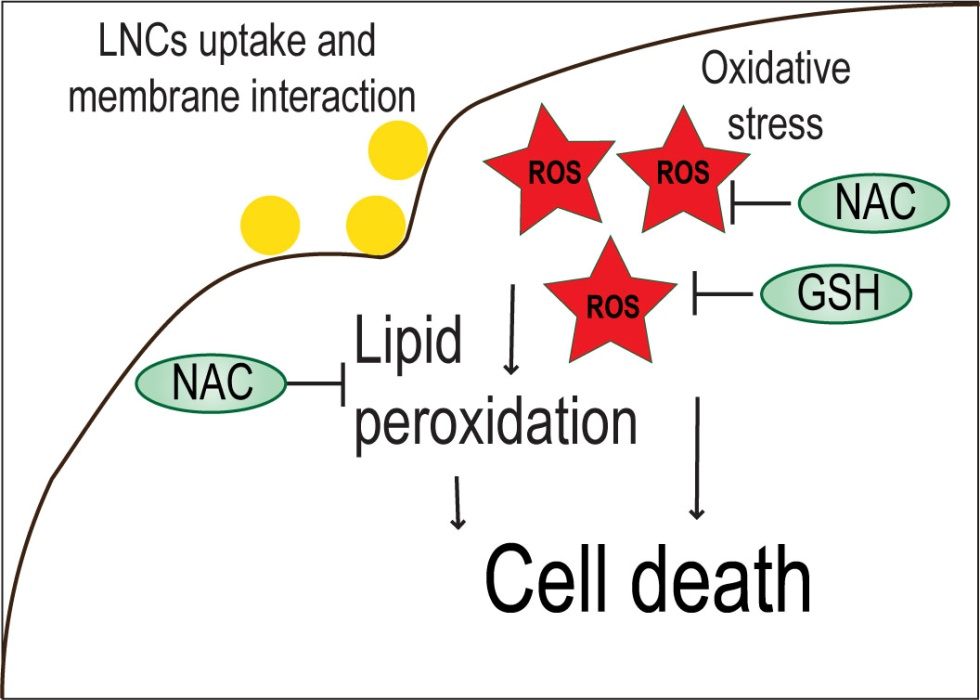
**

**Figure S2.** Overview of changes in the cellular redox homeostasis induced by LNCs.

**
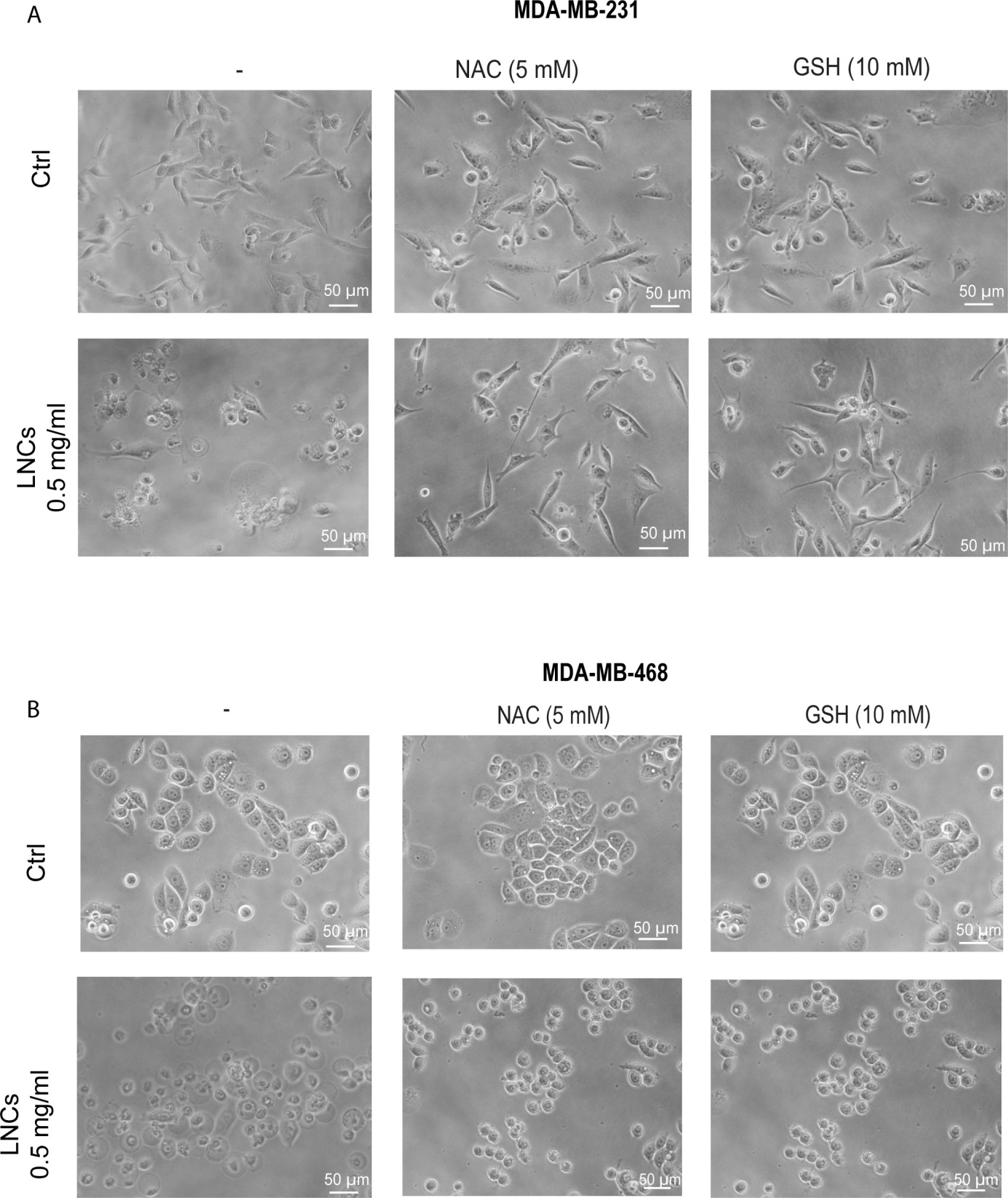
**

**Figure S3.** Micrographs of **a** MDA-MB-231 cells and **b** MDA-MB-468 cells taken after treatment of cells at 37 °C for 24 h with 0.5 mg/ml LNCs in the absence or presence of the antioxidants NAC (5 mM) or GSH (10 mM).

**
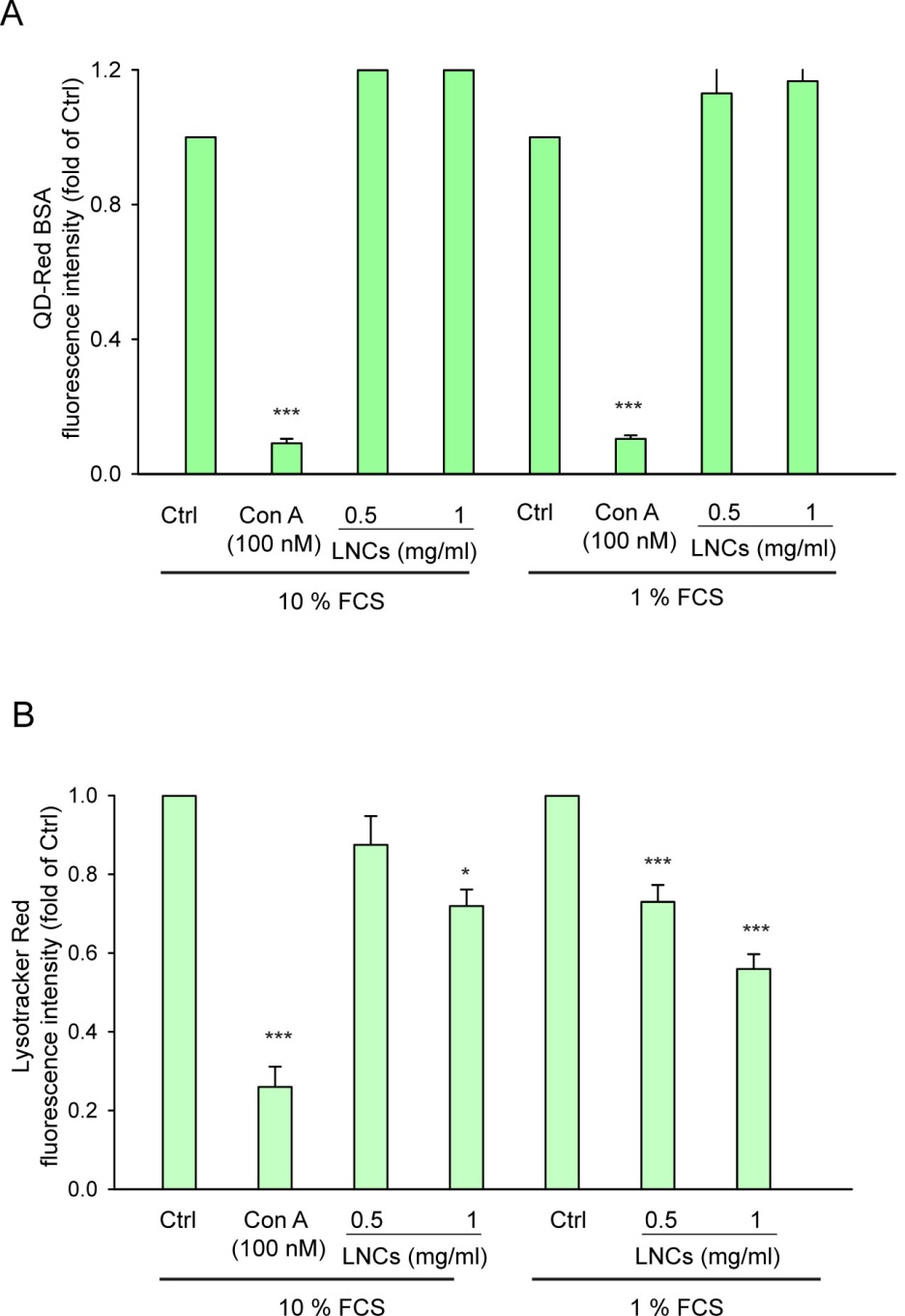
**

**Figure S4. a** Measurements of DQ-Red BSA fluorescence after 2 h incubation with LNCs at 37 °C in MCF-7 cells was performed as described in Fig. 8b, but chasing of DQ-Red BSA was performed in the absence of LNCs. **b** Staining of lysosomes of MCF-7 cells by Lysotracker Red® after 2 h incubation at 37 °C with LNCs was performed as in Fig. 8e, but incubation with Lysotracker Red® was performed in the absence of LNCs.
